# Characterizing stakeholders in cancer primary prevention in European countries: an exploration of challenges and opportunities using a penta-helix framework

**DOI:** 10.3389/fpubh.2025.1550712

**Published:** 2025-07-16

**Authors:** Luis Roxo, Ana Santos, Charis Girvalaki, Marius Geantă, 4P-CAN WP2 study team, Mirjana Babamova, Adriana Boată, Stefania Boccia, Ana Cristina Garcia, Stanimir Hasardzhiev, Andreia Leite, Lilia Kriachkova, Milica Kuzmanoska, Nikola Milasevic, Vlad Nerau, Ivaylo Petrov, Ramona Popescu, Oscar Ribeiro, Viktor Semenov, Leonardo Villani, Ivana Vojvodic, Clara Volintiru, Rodica Milena Zaharia, Serhii Zakharov, Mafalda Sousa-Uva

**Affiliations:** ^1^Department of Epidemiology, National Institute of Health Doctor Ricardo Jorge, Lisbon, Portugal; ^2^Comprehensive Health Research Centre (CHRC), Universidade NOVA de Lisboa, Lisbon, Portugal; ^3^Public Health Research Centre, NOVA National School of Public Health, Universidade NOVA de Lisboa, Lisbon, Portugal; ^4^CINTESIS@RISE, Department of Education and Psychology, University of Aveiro, Aveiro, Portugal; ^5^European Network for Smoking and Tobacco Prevention, Brussels, Belgium; ^6^Center for Innovation in Medicine, Bucharest, Romania

**Keywords:** cancer, Europe, mixed-methods, primary prevention, stakeholders

## Abstract

**Objectives:**

Cancer incidence has been increasing in Europe, with stark disparities between Western and Eastern regions. Cancer primary prevention (CPP) is a cost-effective strategy tackling lifestyle and risk factor exposure, but its implementation goes beyond the actions of the governments. This study aims to characterize stakeholders’ role in CPP, using a penta-helix approach, with the objective of shedding a new light in the Iron Curtain of Cancer Cases.

**Methods:**

We followed a mixed-methods approach, with quantitative and qualitative data from CPP stakeholders from the public sector, academia/research, private sector, media and civil society. Snowball sampling was used to distribute a survey where participants (*n* = 110) were asked which sector was the main driver of change, the most proactive and the most influential. Purposive sampling was used for semi-structured interviews (*n* = 33), where stakeholders were asked about their CPP activities, motivations, barriers and opportunities, and the role of other sectors. Countries were coded as Western or Eastern. Descriptive analysis was used for quantitative data, while thematic analysis was used for qualitative data.

**Results:**

The public sector is viewed as the main driver of change, and the most proactive and influential in both Western and Eastern Europe. However, Eastern European countries emphasize the role of other sectors in CPP more strongly. Thematic analysis identified key roles and themes for the public sector (Strategy: “Looking after citizen’s health,” “Making the system work,” “Operational Engagement”), academia/research (Knowledge: “Scientific credibility,” “Diversity of approaches,” “Getting out of the lab,” “Life in academia/research”), private sector (Responsibility: “Profit-oriented,” “Resources and operational activities,” “Ethics and responsibility”), media (Dissemination: “Capacity to reach people,” “Diversity and scope,” “Information and dissemination”) and civil society (Engagement: “Proximity to people,” “Advocacy and voice,” “Do what others do not do”). Although no meaningful differences were identified between Western and Eastern countries, the results highlight opportunities for Eastern countries to reduce regional disparities.

**Conclusion:**

Overall, results point to the complementary role of the sectors, emphasizing that involving different stakeholders and promoting adequate collaborations between them is crucial to unravel the full potential of CPP.

## 1 Introduction

Cancer remains a major public health concern, associated with significant mortality, disability (1) and economic costs (2, 3). Worldwide, there were close to 20 million new cases and 10 million deaths in 2022 (4), and these numbers are expected to increase over the next decades (5). In Europe, there is still an upward trend in the number of new cases of cancer (6, 7). Cancer mortality rates have been decreasing over time (8), however, progress seems to have slowed in recent years (6, 8).

Significant disparities in cancer epidemiology exist between European countries (8, 9). Eastern European countries exhibit higher mortality rates compared to their Western counterparts (10), reflecting distinct patterns of cancer incidence. Lung cancer is the most frequently diagnosed cancer among males in countries like Montenegro, North Macedonia, and Ukraine, while prostate cancer predominates in most Western European countries. Among women, the incidence of cervical cancer in Bulgaria, Moldova, and Romania is more than twice as high as that in Italy or Portugal (11). Differences in mortality appear to be increasing in relative terms, and are more pronounced for the upper respiratory tract, stomach, bowel, and lung cancers among men, and for uterine cancer in women (10). This West–East divide—*Iron Curtain of Cancer Cases—*may stem from a broad range of social and epidemiological determinants, such as variations in government-led cancer prevention and screening initiatives, exposure to diverse risk factors, differing lifestyle practices, and unequal access to healthcare services (12).

A significant portion of the burden of cancer can be attributed to modifiable factors, such as smoking, alcohol consumption, dietary habits, and exposure to chemicals and environmental pollution (13, 14). This underscores the importance of Cancer Primary Prevention (CPP), which aims to address these factors to prevent the onset of the disease. CPP focuses on reducing exposure to harmful elements and promoting behaviors or conditions that enhance protection against cancer (15). This prevention is achieved through a combination of individual lifestyle changes (e.g., smoking cessation), regulatory actions (e.g., taxing unhealthy beverages), and broader policies at the populational level (e.g., reducing air pollution) (13, 16, 17). Studies have considered CPP a cost-beneficial approach (18), with significant potential to reduce incidence and mortality (5, 19, 20). Moreover, CPP may play a pivotal role in addressing disparities in cancer incidence between countries (8, 9), and social groups (21).

Most countries have approached CCP as part of their National Cancer Control Programs, aiming to reduce cancer burden by defining national priorities and implementing evidence-based strategies. While these programs are heterogeneous, most European countries have national programs tackling tobacco and alcohol use, healthy diets, active lifestyles, vaccination and ensuring safe environmental and occupational conditions (12). Yet, while the effectiveness of these programs has not been thoroughly studied, it is strongly believed that they have not fully realized the potential of CPP (12). CPP must be a collective undertaking, as individual behaviors are deeply intertwined with social, cultural, political and environmental structures (16) and some preventive measures require action at a populational level (22). Active engagement from different sectors of society, such as media, private sector, and civil society, may be required (23).

Understanding the actual and potential roles of these stakeholders is one of the first steps in planning their engagement. The main objective of this study was to characterize the roles of CPP stakeholders from different sectors across Europe, identifying activities, motivations, barriers and opportunities in their actions. The secondary objective of this study was to shed a new light on the Iron Curtain of Cancer Cases, by identifying potential differences between Western and Eastern countries and reflecting on the experiences of Eastern countries.

## 2 Materials and methods

### 2.1 Framework

This study was part of the project “4P-CAN – Personalised cancer primary prevention research through citizen participation and digitally enabled social innovation,” developed by a consortium of 11 European countries (24). This international project uses multidisciplinary resources to understand the determinants of policy implementation and adherence to healthy lifestyles, with the main goal of improving CPP and reducing inequalities in Eastern European countries. In this study, we used a mixed-methods approach to characterize stakeholders, drawing on both quantitative and qualitative data, from a cross-sectional online survey and semi-structured interviews, respectively. We used the penta-helix approach (25), a framework commonly applied to understanding the roles of various stakeholders in innovation and policymaking (26, 27). Our rationale for choosing this framework was twofold: first, it aligned with the 4P-CAN Project’s goal of developing innovative approaches to CPP through multidisciplinary resources; second, this study built on existing knowledge suggesting that CPP requires shared responsibility across different sectors of society, considering multiple interconnected factors (e.g., individual, social, or political). Thus, stakeholders were grouped into five sectors: public governance/public sector (hereafter “public sector”), academia/research, private sector, media, and citizens/civil society (hereafter “civil society”). A description of these five sectors is available in Supplementary Table S1.

To shed new light on the determinants of the *Iron Curtain of Cancer Cases*, consortium countries were divided into two groups: Western (Belgium, France, Ireland, Italy, and Portugal) and Eastern (Bulgaria, Moldova, Montenegro, North Macedonia, Romania, and Ukraine). This classification follows previous research on the West–East division (10). The quantitative part of the study received responses from participants in other countries outside the consortium, which were coded based on geographical proximity and epidemiological patterns (Supplementary Table S2).

### 2.2 Data collection

#### 2.2.1 Quantitative data

This study used data from a larger questionnaire designed to identify CPP stakeholders, characterize their activities and assess CPP performance indicators among consortium countries. This questionnaire was distributed using a snowball sampling method. The 4P-CAN consortium partners were asked to invite at least six national CPP stakeholders to participate in the survey (assuming that an invitation from someone within their own country would encourage higher participation rates). The invitations (containing a link and informative brochure) were sent simultaneously to the stakeholders identified by the consortium partners in January 2024. After completing the survey, participants were invited to forward the link to other CPP stakeholders.

The online questionnaire was created using RedCap 10.9.2 software and was presented in English. Three survey questions were used in this study to evaluate the perceived primary role in change, proactivity, and influence of different sectors. Respondents were asked to rank the sectors (e.g., *Which sector do you consider that could have a role in change concerning cancer primary prevention? Please rank from most important to less important.*). In the analysis, only the sector ranked first by each participant was considered.

#### 2.2.2 Qualitative data

Semi-structured interviews were conducted to gain deeper insights into the specific roles of each sector in CPP. Purposive sampling was employed, meaning that participants were selected based on their expected ability to provide new perspectives (28). Additionally, a stratified sampling approach was used (29), to ensure representation of the five stakeholder sectors of stakeholders across both Western and Eastern countries. The sample size was estimated at 30 (three participants for each sector/country group), depending on participants’ availability, willingness to participate, and saturation of information.

Partners of the 4P-CAN consortium were asked to provide a list of two/three CPP stakeholders from each penta-helix sector, along with a brief description of their main activities. The leading team selected potential participants to ensure a sample with diverse perspectives and experiences. Initial invitations (including an information brochure about the project) were, then, sent by the study partners. To respect confidentiality and protect personal information, the invitation was sent by each partner to their chosen list of stakeholders while the leading team contacted participants only after they had agreed to share their contact details. The interviews were conducted online between July and September 2024, by four interviewers with prior experience in health-related semi-structured interviews.

The interviews were conducted using a structured guide that was divided into four sections: (A) Introduction and characterization of interviewee (e.g., *“In which sector are you involved?”*), (B) Activities (e.g., *“In which main CPP activities are you involved?”*), (C) Perceptions about other sectors (e.g., *“For each sector, could you please tell us about the main differences in their activities and motivations?”*), and (D) Interaction between stakeholders (e.g., *“with which main partners do you collaborate when you develop CPP activities?”*). A complete list of questions can be found in Supplementary Table S3.

### 2.3 Data analysis

#### 2.3.1 Quantitative data

Relative frequencies (%) were computed for each variable and are presented for the total sample and by country group (Western and Eastern). Statistical analysis was performed using R software.

#### 2.3.2 Qualitative data

Interviews were coded into four categories: (1) Activities (description of specific activities, how the activities are structured, the players involved, or the scope of action of each sector), (2) Motivations (description of reported motivations or underlying ideas driving the actions of each sector), (3) Barriers (description of reported barriers or evaluation of a sector’s actions) and (4) Opportunities (description of reported suggestions for improvement, potential role, and activities not yet developed for CPP). These four categories were selected to offer a clear understanding of the current and potential roles of CPP stakeholders, with the goal of addressing the main goal of the study. Thematic analysis was performed to identify common and relevant patterns of meaning for each sector (30), overlapping these four categories, while considering the complexity and interconnectedness of ideas across them.

Some participants described activities, motivations and barriers related to cancer secondary prevention or treatment. These were included when they were indistinguishable from CPP (e.g., when describing motivations). When these descriptions revealed possible links, ideas and collaborations relevant for CPP (e.g., awareness campaigns about screening), they were categorized as “opportunities.”

The first two interviews for each sector/country group were fully transcribed and a grid of analysis was filled with quotes regarding the activities, motivations, barriers and opportunities for the different sectors. Initial codes were generated using a *bottom-up* approach. Themes were gradually and iteratively formulated through continuous joint reflection between the interviewers. The final interviews were only partially transcribed and reviewed for new information. Here, a *top-down* approach was taken, using the previous structure and adding codes only when new ideas were introduced. This approach was adopted after reaching data saturation, as only minor information was being added to the interpretation (31). Finally, for each sector, a general word summarizing the overarching themes was identified to encapsulate the central idea conveyed by the interview responses. To enhance readability, each theme is described narratively, providing a more cohesive and fluid presentation of the findings while addressing overlapping information across categories. This description is supported by a list of topics organized by theme, category, and sector in tables, along with selected quotes provided in Supplementary Tables S4–S9.

### 2.4 Ethics statement

Participation in both the survey and interviews was voluntary. Study partners distributed invitations, which included an information brochure outlining the project’s objectives, data handling procedures, and informed consent process. The survey did not collect any personally identifiable information. Consent for recording the interviews was obtained at the beginning of each interview. All data were anonymized and handled confidentially by the leading research team. No personal or identifiable information was shared with study partners or third parties to ensure participant privacy and compliance with ethical standards.

## 3 Results

### 3.1 Survey results

A total of 110 stakeholders responded to the survey. Of these, 56.4% were from Western countries. Approximately half of the participants (49.1%) were from the public sector, followed by 20% from academia/research, 23.6% from civil society, 4.5% from the media, and 2.7% from the private sector.

**Figure 1:**
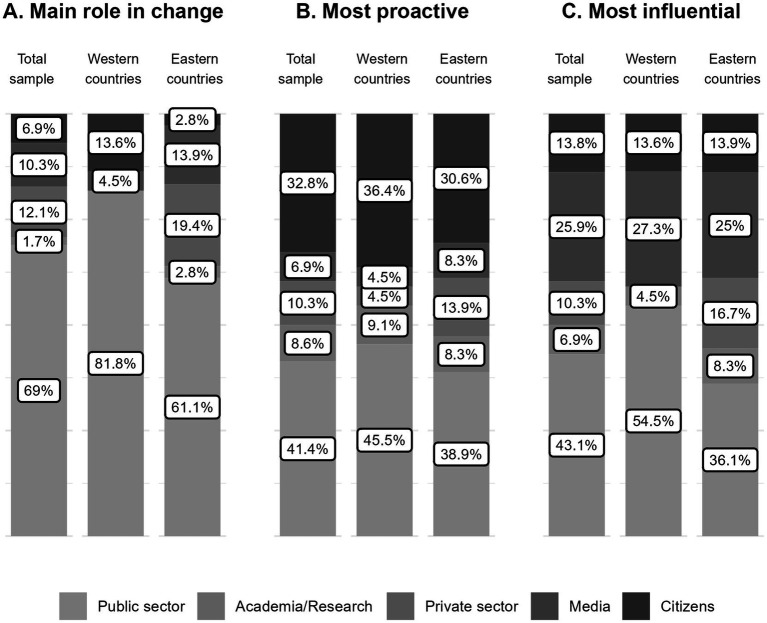
Results from the survey to CPP stakeholders. Percentage (%) of participants identifying each of the five sectors (public sector, academia/research, private sector, media, and citizens/civil society) as having the main role in change, being the most proactive, and the most influential, shown for the total sample, and for respondents from Western and Eastern European countries.

For the overall sample, the public sector was widely regarded as the key driver of change, with 69% of all participants identifying it as the sector with the main role (Figure 1A). This trend was observed in both Western and Eastern countries. In Western countries, a larger proportion (81.8%) prioritized the public sector, followed by civil society (13.6%) and the media (4.5%). In Eastern countries, 61.1% of respondents identified the public sector as the primary driver of change, followed by the private sector (19.4%), the media (13.9%), and, in smaller proportions, civil society and the private sector (2.8% each).

The public sector and civil society were most frequently recognized as the most proactive sectors (41.4 and 32.8%, respectively) across the total sample (Figure 1B). This pattern was consistent in both Western (45.5 and 36.4%) and Eastern countries (38.9% and 30.6%). Among participants from Western countries, only 18.1% identified other sectors as the most proactive. In Western countries, the remaining 30% of responses were distributed among the private sector (13.9%) and the media and civil society, each at 8.3%.

Similarly, the public sector was found to be the most influential sector (43.1%) across the overall sample (Figure 1C). More than half of participants from Western countries (54.5%) rated the public sector as the most influential in their countries, compared to only 36.1% of those from Eastern countries. The media was the second sector most frequently considered the most influential by both Western (27.3%) and Eastern (25%) respondents.

### 3.2 Interviews results

**Figure 2:**
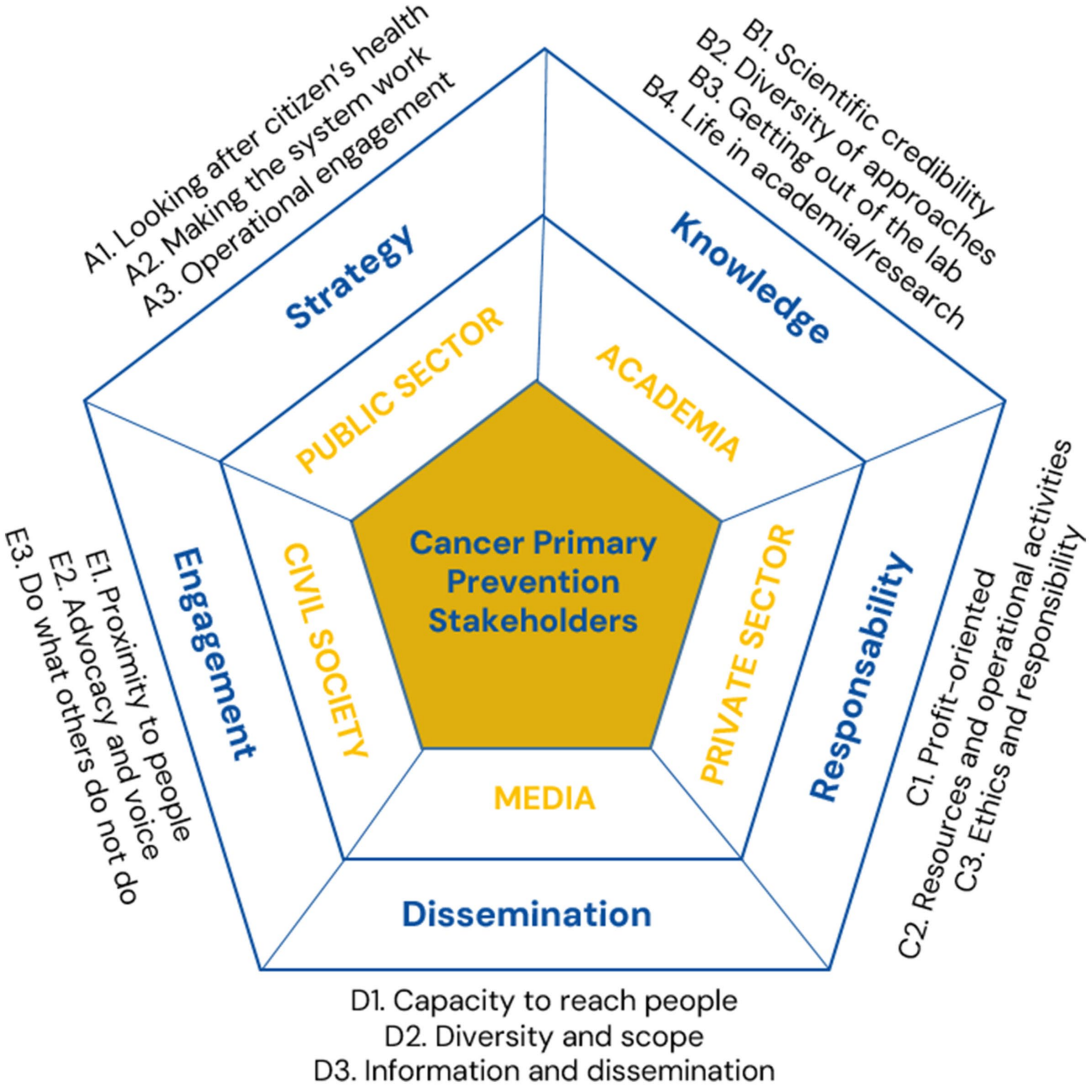
Themes and overarching concepts identified in the qualitative analysis for the five sectors (public sector, academia/research, private sector, media, and civil society).

In total, we conducted 33 interviews with CPP stakeholders, 12 with participants from Western countries and 21 with participants from Eastern countries (Supplementary Tables S10, S11). Since few persons appeared to be exclusively dedicated to CPP, it was difficult to recruit participants, particularly from the media. Participants from Eastern countries seemed more willing to participate than their Western counterparts. The thematic analysis of the interview data allowed us to identify key themes of each penta-helix sector regarding their activities, motivations, barriers, and opportunities (Figure 2).

#### 3.2.1 Public sector

The word *“Strategy”* was identified as the overarching concept for the public sector, with three themes identified: *“Looking after citizen’s health”*; *“Making the system work”*; and *“Operational Engagement”* (Table 1).

**Table 1:** Results from thematic analysis of the interviews.

Activities	Motivations	Barriers	Opportunities
A1. Looking after citizen’s health			
Complexity of players Power Strategic role	Responsibility and impact Tackle burden of disease	Political circumstances Threats to power Prevention not a priority Institutional barriers	Pivotal role in change Have a wider scope Gains in the long term Cooperation/involvement Political opportunities Become more aware
A2. Making the system work			
Epidemiology Study what works Provide structure	Improve the system	Structural issues Insufficient data	Create networks of professionals Write procedures International cooperation
A3. Operational Engagement			
Health education Do screening programs	Educate the population	Lack of money People-related barriers Difficulties implementing Inequalities Human resources	Deal with hierarchy Extend the scope of activities

List of main activities, motivations, barriers and opportunities, among the public sector (A1–A3).

***Looking after citizen’s health***. Many participants described the public sector’s main responsibility for ensuring health and well-being of the population, by addressing the burden of cancer in society and investing in the long-term benefits of CPP (“*because of their essence, of their core business, that is to protect the public, the population, from harmful behaviors, harmful products”*).

The public sector was often described as having an influential and important role, with financial resources, the authority to allocate them, and the legal power to produce change. Some of the indicated barriers are linked to limitations in the public sector’s power, such as the inability to counteract industry lobbies. Furthermore, the public sector involves a highly complex network of players, including local, regional, national and international institutions, which at times experience difficult interactions, including challenges in communication and information sharing.

The definition of strategies (e.g., national cancer plans) was also recognized as a responsibility of the public sector, although several participants criticized the lack of a clear strategic role. While setting the agenda, CPP (or even health in general) is often not prioritized compared to cancer treatment or other diseases. As policymaker, the public sector was also acknowledged as responsible for developing policies and regulations to tackle modifiable factors, such as through taxation.

The public sector’s close ties to politics were frequently highlighted. CPP is often impacted by political instability or changes in government, leading to disruptions of previous initiatives. Conversely, new political cycles can present opportunities to launch new activities. As some politicians are not from the health sector, they may lack awareness of the relevance of CPP. Moreover, CPP does not seem to be a topic that generates political capital, as the effects are not visible in the short term, and unpopular decisions may be needed (e.g., product bans).

***Making the system work***. Another facet of the public sector’s role in CPP is to provide the structure that makes the health system functional, focusing on its continuous improvement. Some participants reported that the public sector is involved in health surveillance and epidemiological research, providing and collecting data (*“we did not have any information about incidence and survival. That’s why we started this population-based cancer registry […]. And right now, we have all these key metrics, survival, prevalence, mortality, and incidence”*). This extends to modeling the impact of policies, assessing and piloting new interventions, and considering the sustainability of the health system. Other activities include consulting experts, creating networks of professionals, and developing information technologies. Herein, participants reported structural barriers within the public sector that affect its capacity to address cancer (e.g., difficulties achieving universal health care). One participant suggested writing procedures, while international cooperation is seen an opportunity, using tools from EU or following examples from comparable countries.

***Operational engagement***. The public sector plays a vital role in a wide range of operational activities, directly engaging with communities and addressing their immediate needs (*“after that, the Ministry of Health is only operational”*). Participants mentioned that the public sector is involved in activities such as screening, health education, compiling scientific information, or building facilities for physical exercise. Some participants stated that translating policies into practice is difficult and too indirect, with the scarcity of financial and human resources often being mentioned. Implementation efforts also face challenges related to the population, such as low educational levels, inequalities between groups and difficulties in reaching disadvantaged populations. New initiatives were suggested, such as increasing the focus on health education in schools and oncology hospitals, or organizing sports sessions. One participant stated that, when dealing with these operational tasks, support from lower-level hierarchies is crucial for success.

#### 3.2.2 Academia/research

The word *“Knowledge”* was identified as the overarching concept for academia/research, with four themes being pointed out: *“Scientific credibility”*; *“Diversity of approaches”*; *“Getting out of the lab”* and *“Life in academia/research”* (Table 2).

**Table 2:** Results from thematic analysis of the interviews.

Activities	Motivations	Barriers	Opportunities
B1. Scientific credibility			
Trustworthiness Knowledge creation Scientific support to others’ activities			
B2. Diversity of approaches			
Basic science Characterize the situation Project evaluation Innovation	Raise awareness to a difficult situation Lack of data	Financial barriers Human resources No clear direction No resources/no equipment Limited scope	Expand the scope of research Multidisciplinary/integration International cooperation Increase support resources
B3. Getting out of the lab			
Teach/train Write guidelines Education at schools	Empower people Get down the cancer/current state	Isolation/no collaboration	Make research more visible Propose policies Do training for other sectors
B4. Life in academia/research			
Academic cooperation Step in the career of people from other sectors Meetings/networks	Scientific interest Get funding Contamination between areas	Methodological aspects Journals not interested Difficulties finding financing Focus on metrics	Leave the bubble

List of main activities, motivations, barriers and opportunities, among academia/research (B1–B4).

***Scientific credibility***. The ideas of credibility and trustworthiness were often used to describe the activities of academia/research. Information from academia/research is generally accepted, and researchers are regarded as sources of authority. One of the goals of academia/research is to build knowledge on cancer and CPP, to understand the current situation and to explore potential solutions. To achieve this, they rely on robust methods, previous evidence *(“looking at the prevalence of cancer and how at a science, biological, medical, physiological, psychological, behavioral aspect that we can target cancer, but also prevent the prevalence of cancer”)*, and the search for scientific consensus. This information can inform other stakeholders, influencing the activities of other sectors. For instance, the public sector may rely on research evidence to design policies, while civil society may use data to support their advocacy efforts.

***Diversity of approaches***. Participants described a wide range of activities (*“we have different groups that are trying to do this, from different perspectives”*), from the characterization and evaluation of projects to innovative approaches to prevention. Some participants noted the lack of previous data and are conducting epidemiological research to raise awareness of CPP. Other participants combine their activities with basic science research. Additional research activities include exploring innovative ways to disseminate messages that promote change, such as using apps or TV shows. Notwithstanding, the scarcity of financial and material resources, along with the overload of human resources, hinders the development of some activities. Some participants mentioned the need to broaden the scope, noting a limited number of activities and research groups. A clear definition of research priorities also seems to be missing, and the lack of data hinders the achievement of goals. Collaboration between institutions presents an opportunity, particularly cooperation between different sectors and disciplines, both nationally and internationally.

***Getting out of the lab***. Some participants described how researchers can play an active role outside research *(“get out of the lab, go out in the street when you do fundraising and talk about these things as well”)* and how academia/research can contribute to implementing its findings. Some participants described teaching and participating in the training of health professionals, while others are involved in community health education activities. Researchers can also create resources for other sectors, such as educational programs or guidelines. Some researchers are motivated by the goal of educating and empowering the population, using existing knowledge to address the burden of cancer and the strain on healthcare services. However, efforts to engage with the community can be hindered by researchers’ isolation and challenges in collaborating with other stakeholders. The possibility of proposing new policies and initiatives and providing training for persons from other sectors is seen as an opportunity.

***Life in academia/research***. Some descriptions of academia/research participation in CPP are specific to how this sector works. Holding conferences, networking, consulting for other research projects, and cooperating with other universities or research centers were mentioned as activities. Academia/research is often a career step of people from other sectors. For instance, one participant from the public sector was pursuing a Ph.D. about cancer screening, while others try to integrate research into their professional activities. Scientific curiosity was described as a motivation, as well as invitations from other researchers and the evolution of personal scientific interests *(“I studied the microscopic lesions of these tumors in cows. And I gained a special interest in oncology after this”)*. Participants also described several methodological challenges in CPP research, noting that it requires long-term studies and is expensive. One participant described the difficulties of building a career in research, while another mentioned that scientific journals are not particularly interested in this topic. The difficulty in securing funding was often reported, exacerbated by the challenge of fitting CPP into existing financial calls, and the lack of interest from funding agencies. Conducting research is easier when financial support is not required. One participant described how academia/research often shifts its focus and structure based on available funding.

#### 3.2.3 Private sector

The word *“Responsibility”* was identified as the overarching concept for the private sector, with three themes: *“Profit-oriented”*; *“Resources and operational activities”*; and *“Ethics and responsibility. (Table 3)”.* While several participants from other sectors reported that the private sector is not interested in CPP, participants from this sector often perceived it as very proactive and having large potential.

**Table 3:** Results from thematic analysis of the interviews.

Activities	Motivations	Barriers	Opportunities
C1. Profit-oriented			
Development of products/innovation Activities dependent on their objectives	Profit Limited interest Marketing and image Own agenda	Focus on profit Insufficient involvement Lack of return Limited scope/inequalities	Clear message/build their reputation Building the relationship/Red lines Regulations
C2. Resources and operational activities			
Providing resources Support others’ activities Raise awareness Vaccination/screening Capacity to reach people	Educate and promote health of people/change mentalities	Bad reputation Conflict of interests People’s beliefs	Expand activities/think outside the box. Geographic and sociodemographic outreach of pharmacies Pharmacies’ collaboration with research
C3. Ethics and responsibility			
Promote the health of the employees (included in general health promotion)	Personal reasons Capacity to prevent cancer Ethical concern/responsibility	Pressure on governments, pressure on citizens	Be responsible selling products Companies as a concentration of people

List of main activities, motivations, barriers and opportunities, among private sector (C1–C3).

***Profit-oriented***. Profit was often seen as a primary motivation for the private sector *(“We have to balance what we do probono and what we have to do to pay salaries!”)*, significantly influencing their participation in CPP initiatives and the scope of their activities. For example, pharmaceutical companies may focus on just one type of cancer, or private companies may be less inclined to engage if there is limited financial benefit. In some cases, the private sector’s engagement is perceived as driven by marketing objectives or attempts to improve public image, often with limited substantive impact. This profit-driven approach also drives investments in innovation and the development of new products. Some participants reported that this may create conflicts of interests, a challenge that some countries address through strict regulations. Other participants emphasized that successful collaboration with the private sector requires building trust and setting clear boundaries.

***Resources and operational activities***. The private sector was recognized for its resources and its potential to support other sectors *(“funded either specific projects, specific research activities, specific communication activities, targeting CPP”)*. Many private companies contribute to CPP by developing and promoting awareness campaigns aimed at empowering individuals and changing societal attitudes, or by engaging in operational activities, such as vaccination and screening initiatives. These resources are also used to enhance public outreach and fund projects led by other sectors. However, private sector activities often face limitations due to factors such as bad reputation, regulatory constraints, or varying contextual priorities. Despite these challenges, private sector representatives expressed a willingness to expand their contributions to CPP. Suggested initiatives included increasing educational efforts targeting children, collaborating with cancer survivors, addressing hereditary risk factors, and combating societal stigma surrounding cancer. It was noted that stakeholders from other sectors might raise concerns when accepting funding from the private sector. One participant mentioned that projects funded by the private sector can have broader scopes compared to those funded by agencies, which typically prioritize specific tasks and measurable outcomes.

Pharmacies, as part of the private sector, were also acknowledged for their role in operational activities (e.g., smoking cessation initiatives). They bring unique strengths, including broad geographical reach, the ability to connect with diverse sociodemographic groups, and to collaborate with other sectors (e.g., help with research).

***Ethics and responsibility***. Participants from the private sector may engage in CPP activities due to ethical concerns, personal motivations, and the potential to mitigate the burden of cancer (both current and predicted) by using existing knowledge. Some participants reported that the private sector could play a major role in driving change, considering the potential negative impacts of the products they sell. This view was further supported by descriptions of certain companies holding positions of power, which makes regulation harder, and increases the potential for exerting pressure on the public sector and citizens *(“They put pressure on the government by pressuring citizens […] with all sorts of arguments, such as that they help the economy or they start with saying that their products are not harmful, that people should be free to choose”)*. A stronger regulation on advertising presents an opportunity for change.

The role of private companies in promoting the health of their employees was extensively described by one participant, who noted that reaching people through their employment offers a unique opportunity to access a broad audience. This company engages in regular information campaigns promoting healthy diets, physical activity, and screening activities. Such efforts are seen as opportunities to foster healthy habits that employees may carry into their homes and share with their families. This idea was repeated by other participants, who highlight that employers can offer incentives to support cancer prevention.

#### 3.2.4 Media

The word *“Dissemination”* was identified as the overarching concept for the media sector, with three key themes emerging: *“Capacity to reach people”*; *“Diversity and scope”*; and *“Information and dissemination (Table 4)”*. Most participants described the media sector as important and influential. Its power and potential in CPP were widely recognized, although several participants suggested that more action could be taken.

**Table 4:** Results from thematic analysis of the interviews.

Activities	Motivations	Barriers	Opportunities
D1. Capacity to reach people			
Great impact Place to influence people/policy Create awareness	Pressure policies Public service Dubious motivations Commercial interests	CPP not a priority Influence by politics Compliance with news cycles Bad reputation Conflicts of interest	Powerful tool Adjust the dialog Choose the best channels Impact policies Two sides of the story Public perception change
D2. Diversity and scope			
Different channels, including new media Diversity of topics and activities		Channels not trustable Sources not trustable Online commentaries Lack of in-depth focus Too many scopes	Visibility given by new media
D3. Information and dissemination			
Provide information Disseminate specific news Interaction with other sectors	Educate the public Responsibility Personal experiences Interesting topic	Responsibility not fulfilled Unprepared journalists Low quality of information Lack of resources	Adjust the messages Send the right messages Training for journalists

List of main activities, motivations, barriers and opportunities, among media (D1–D3).

***Capacity to reach people***. Media’s presence in people’s everyday life contributes to its influence and impact *(“There is no month without a cancer debate on different subjects, treatment, education, screening, prevention. And I think because of this discussion, media can send a lot of messages and they are very influential, because we cannot reach so many levels like the media”)*. This includes reaching persons who have not been diagnosed with diseases, who may be less aware of cancer and could benefit the most from CPP. Media can affect people’s health behaviors and change public perceptions, as part of their role includes raising awareness of the benefits of CPP. Pressuring political entities and helping the search for solutions were reported as motivations. Despite this, several participants mentioned that CPP (or health in general) is not a priority for the media. Coverage is often superficial, aired in unattractive times, and is frequently overlooked by the news cycle. Sometimes, interest in covering CPP is restricted to specific events, or shocking news. Suggestions were made to extend coverage beyond awareness months and create messages that are more attractive to the media.

While reaching a large number of people, media faces other barriers such as conflicts of interest, political interference and bad reputation, which may hinder collaboration with other professionals. Listening to different perspectives may help in addressing these issues. Some participants reported difficulties in trusting professionals and feeling pressured to comply with their narratives. Still, this relationship can be built gradually over time.

***Diversity and scope***. Participants reported a wide variety of media channels (e.g., television, blogs, podcasts), allowing the implementation of different activities, ranging from debates and interviews with specialists and researchers to messages aimed at increasing CPP awareness, and scientific news targeting health professionals. Some participants mentioned more innovative ways to disseminate messages, such as a fictional TV show about cancer prevention or creative content on social media. This diversity makes it possible to reach people from different sociodemographic groups (*“Depending on the target population, depending on the age, some people listen more to radio, some other to television, some other do not trust the radio or television, but they go to the stadium”*), although one participant noted the absence of messages on online platforms directed at young people. Managing the messages sent through different scopes, from national to supranational contexts, can be complex.

A special focus was placed on new online media channels. These may increase the visibility of CPP but raise significant concerns about the quality of information. Social media has been described as an appropriate way to share stories, and influencers and celebrities can boost publicity. However, due to stigma, some celebrities may fear being associated with cancer, while others may have profit-driven goals. Moreover, new challenges need to be tackled, such as online comments.

***Information and dissemination***. Information can be rapidly shared by media (*“I can have a press release and be read by one thousand, two thousand citizens, but the news in the media can go to a hundred thousand millions”*), and it can be stored and accessed at any time. This capacity can be used to disseminate particular news, such as the launching of national cancer plans, or official campaigns from health authorities. Furthermore, media can spread general ideas about health promotion and share information about the activities carried out by other sectors. The language style must be simple, understandable, non-alarming, and reliable.

Low quality of information is a relevant barrier when describing media’s actions, especially (but not exclusively) regarding new media channels. Some journalists are not aware of the importance of CPP and lack the knowledge to share accurate information or ask adequate questions. This issue can be addressed through specific training, or by providing brief explanations before press conferences. In some cases, communication is handled by non-specialized human resources, such as doctors. Some participants highlighted that the media can facilitate awareness campaigns and help restrict advertisements for unhealthy products.

#### 3.2.5 Civil society

The word *“Engagement”* was identified as the overarching concept for civil society, with the following themes: *“Proximity to people”*; *“Advocacy and voice”*; and *“Do what others do not do”* (Table 5).

**Table 5:** Results from thematic analysis of the interviews.

Activities	Motivations	Barriers	Opportunities
E1. Proximity to people			
Participation of persons from other sectors Personal involvement Proximity to people	Personal involvement besides one’s job Personal experience Change public perceptions	Lack organization/lack strength/lack power Disregard by others No expertise Stigma/cultural issues People-related barriers	Changes in structure Joining forces Advocacy/strength to change things
E2. Advocacy and voice			
Influence and advocacy Personal history as their voice Central part in the health system	Burden of cancer Raise awareness Inefficiency of the government	Lack of awareness Insufficient involvement/not a lot of patient organizations Isolation/no visibility	Expand activity International cooperation Fill the needs Opportunities to educate/raise awareness
E3. Do what others do not do			
Education/awareness Filling the gaps Extensive scope of activities		No defined strategy Lack of resources Limited scope Overlapping services	

List of main activities, motivations, barriers and opportunities, among civil society (E1–E3).

***Proximity to people***. The idea of proximity was highlighted when describing civil society (*“They cannot influence directly how people behave and how people can change their habits. And I think civil society, citizens have that role”*), a sector that often uses flexible methods that more easily connect with people, bridge policies and individuals, and drive change. This was also reflected in the motivations of people from this sector, many of whom have personal or familial experiences with cancer. Some participants from other sectors also volunteer in civil society organizations, while one participant has taken on multiple job roles in various sectors, driven by a personal interest in CPP. Another participant expressed a will to engage in CPP in their private life, in addition to their work in the private sector. Proximity to individuals and communities allows civil society to engage with people directly but also exposes them to various personal and cultural challenges, such as low educational levels, stigma, and dominant societal values. On the other hand, a lack of expertise can hinder the ability to define priorities and maximize the sector’s impact. Civil society may also face challenges like too informal approaches, lack of organization, and limited power, leading to its potential disregard by stakeholders from other sectors.

***Advocacy and voice***. Civil society plays a central role in CPP and the health system, driving significant change. The voice of civil society can communicate particular needs (*“it is important that patient representative must sit in the government, as a representative of patients, to share their voice and their opinions and their needs”*), push agendas, change public perceptions, influence the allocation of resources, inform particular policies and help implement them. Some participants described this strong voice as lobbying. Particularly, the perspectives of cancer survivors seem to have a great influence and can serve as examples for other citizens.

On the opposite, some civil society organizations may not yet fully recognize the importance of prevention or may prioritize treatment over prevention. Some participants from other sectors reported that civil society’s involvement is insufficient or too isolated, with too few organizations or a lack of visibility. The work of civil society could be enhanced by developing partnerships, creating federations, and investing in human resources.

***Do what others do not do***. Civil society is involved in a wide range of activities, often filling gaps left by other sectors (*“I think the civil sector does everything that the other sectors do not do”*). These activities include raising awareness, sensitizing, and empowering the population. This may involve visiting schools to reach young people with tailored messages that can have a stronger impact than those from their families. Other activities include creating networks to foster collaboration between doctors and experts, organizing screening programs and seeking fundraising. Some participants from this sector also reported involvement in European projects, with the potential for connecting with patient organizations from other countries highlighted. The motivation to engage in these activities often stems from recognizing the burden of cancer and the perceived inefficiencies in the implementation of governmental measures. However, the lack of defined strategies has been described as a barrier.

The scope of activities for civil society could be expanded, for example, by distributing healthy food, focusing on multiple cancer risk factors, or reaching more geographical areas. However, the lack of resources, particularly financial resources, hampers the ability to implement additional activities. One participant mentioned the challenge of reaching those who could benefit most from public health programs, while another noted some overlap with services provided the municipalities. Several opportunities to broaden the activities of civil society were identified, such as organizing more events, becoming more involved in policymaking, or educating journalists on the importance of CPP.

#### 3.2.6 Iron curtain of cancer cases

The qualitative thematic analysis did not uncover meaningful differences between Western and Eastern countries (*“the level of the motivation or the level of obstacle* var*ies between countries, but the obstacles and the motivation are pretty much the same”*). Yet, participants from Eastern Europe highlighted certain barriers and opportunities that could be addressed to reduce regional disparities.

Participants from Eastern European countries acknowledged the relevance of CPP and its potential to save resources and address the growing burden of cancer. However, meaningful challenges remain, including limited government interest, a lack of strategy, and insufficient long-time planning. Additionally, participants highlighted the absence of cooperation between government agencies, academic institutions, and NGOs, with no shared vision between them. This hinders the collaborative efforts necessary for effective CPP implementation.

Public sector actions are often deprioritized due to financial constraints, frequent governmental changes, and political instability, all of which disrupt the continuity of health initiatives. This environment leads to outdated practices, policies, and laws, often lacking data-driven support. Despite participants recognizing the value of epidemiological data for policy advocacy, such data appear to be insufficient or missing. While the private sector, media, and civil society may help fill some gaps (e.g., raising awareness for CPP), the absence of a clear strategy remains a challenge. Additionally, cultural issues and stigma further limit citizen engagement with CPP.

The small size of some countries also affects the scope of activities pursued by the different sectors. For instance, one participant working on the pharmaceutical industry mentioned that their company lacks a representative office in the country, and that makes them more reliant on external entities. Similar challenges are observed in civil society, where there are reports of only a few patient organizations and difficulties in forming a united front. International collaboration was often seen as an opportunity, whether through EU membership, participation in EU-funded projects, or adopting successful practices from other countries.

## 4 Discussion

This study combines quantitative and qualitative data to characterize the roles of stakeholders from the public sector, academia/research, private sector, media and civil society in CPP, while also exploring potential differences underlying the *Iron Curtain of Cancer Cases*. Quantitative results indicate that the public sector is still considered the key driver of change, the most proactive and the most influential, but this result seems less pronounced in Eastern than in Western European countries. Qualitative results reinforce that the five sectors have distinct yet complementary roles in CPP. While motivations and barriers appear similar among Western and Eastern countries, data show relevant opportunities for Eastern countries.

Key findings of this study suggest that the public sector is often expected to provide strategic direction and structural support, with a noticeable gap when this role is not fulfilled. This aligns with previous literature emphasizing the need for clear and committed leadership in addressing non-communicable diseases (23). The public sector can implement measures such as legislation, regulation, enforcement, voluntary guidelines, incentives, and education campaigns to reduce cancer risk at the population level (15). Public sector action is particularly relevant in cases of involuntary exposure to pollutants or radiation but can also extend to risks associated to individual choices (e.g., regulating tobacco sales) (15). A critical aspect of the public sector intervention is mitigating commercial influences on health. Described as the ways in which commercial entities influence public health, often through marketing, regulatory lobbying, and supply chain practices (32), the commercial determinants of health have gained increasing attention (33). Governments must regulate the tobacco, alcohol, and ultra-processed food industries, as these sectors contribute significantly to cancer incidence. However, industry lobbying can undermined these efforts (32).

Participants emphasize the connection between the public sector, politics, and CPP, noting that politicians may lack awareness, show little interest, or fail to ensure continuity during periods of political transition or instability. Public health prevention measures often have a paradoxical nature, as they are widely supported by politicians and the public, but have low visibility, operate behind the scenes, and are successful precisely when they prevent an outcome from occurring (34, 35). Despite their importance, prevention efforts often fail to generate political capital, as their benefits are less tangible and immediate than those of other policy areas. Future research could explore this further by investigating, for example, potential associations between political instability (e.g., government changes) and CPP indicators. Additionally, qualitative research involving politicians could provide insights into how to build consensus and elevate CPP as a political priority.

Academia/Research can generate important knowledge to guide CPP and support the activities of other sectors, such as quantifying risks associated with certain exposures and testing CPP interventions. However, this field seems underdeveloped (18, 36), and faces specific challenges. Research on this area can be expensive and methodologically demanding, with non-immediate outcomes and difficulties in quantifying the costs of CPP interventions. As CPP requires multidisciplinary action, new collaborations across different disciplines and community services may be necessary (35). Although CPP is pivotal for decreasing the cancer burden, studies on its cost-effectiveness (18) and systematization of economic evidence information lag behind those in other areas of the cancer continuum (37). Quantifying the benefits of CPP interventions may be crucial for informing politicians about potential savings and raising awareness in society by analyzing outcomes easily understood by the general population (e.g., gains in life expectancy). Moreover, only a small portion of CPP studies focus on implementation (36), highlighting the need to better translate scientific knowledge into practical actions that can drive real-life change. Funding for CPP has been undervalued, with interest from only a few funding agencies (36). Results from our study show how CPP research may be difficult to align with existing financial calls, highlighting the need for more targeted funding opportunities to support this critical area. Furthermore, research plays a crucial role in combating misinformation, which may also be influenced by corporate actors, through industry-funded studies, misleading marketing claims, and selective reporting (32).

The results indicate divergent perceptions regarding the private sector’s role in CPP. Although CPP initiatives may face resistance from the private sector due to conflicting commercial interests (35), our results support that companies from different industries could play both direct and indirect roles promoting and implementing these initiatives (33). Regarding cancer, influential industries such as the tobacco, alcohol, and processed food sectors drive the production of unhealthy commodities, wielding significant economic and social power (33). Participants in our study highlight several key issues related to the commercial determinants of health: their impact on public perceptions through biased arguments, pressures over policies, the use of social responsibility as image-washing, financial power, the impact of regulations, and the importance of developing trustworthy relationships. These key commercial influences have also been highlighted when discussing commercial determinants of health, namely, the aggressive advertising campaigns of some industries, the efforts to undermine government regulations and taxation, and the use of legal challenges to weaken public health legislation (32, 38). Addressing these determinants may require a shift in power dynamics and robust multi-sectoral collaboration (38), ranging from responsible industry engagement to strong governmental policies, such as implementing regulations against predatory marketing practices (33, 38). As a portion of cancer cases is linked to occupational factors, ranging from dangerous exposures (13, 39) to employment arrangements (e.g., shift work) (40), improving workers’ conditions, ensuring their safety, and investing in health promotion initiatives are additional ways the private sector can contribute to reducing the cancer burden. Furthermore, private companies can provide both financial support and non-financial resources, such as expertise and visibility. The provision of resources has recently been identified as one of the pillars of multi-sectoral action against non-communicable diseases (41).

Improving health literacy is an important aspect of CPP (15, 42), as previous research has shown that the public knowledge about cancer and its prevention is low (42, 43). Media can play a crucial role in raising awareness, fostering public pressure, and motivating individual behavior change (44). Participants emphasize the role of new internet media, including *influencers* and social networks. Social media has the potential to disseminate information about modifiable factors for cancer prevention and can serve as a platform for cancer survivors or caregivers to share their stories and discuss ideas to shape public perceptions and influence policies (44–46). The diversity of social media platforms allows for message adaptation and the ability to reach different social groups (47). However, there are concerns about the quality of information available on these new channels. As social media algorithms prioritize sensational and shocking content, even individuals who do not actively seek misleading information are frequently exposed to it (48). Platforms should prioritize credible sources, de-emphasize controversial content, and enforce transparency regarding information sources and sponsorships (49), while new computational technologies may also enhance the monitoring of falsehoods. Participants highlighted training journalists on the relevance of CPP as an opportunity, which has also been noted in the literature, as well as regulating misleading health claims in digital advertising (38). Other possible approaches include developing individual-focused tools such as the European Code Against Cancer, whose 4th edition presents 12 evidence-based, easily communicated recommendations to reduce cancer risk (50). These recommendations are designed to be easily understood by both journalists and the general public.

Past experiences highlight the potential of civil society to lead multi-sectoral initiatives, sparking change by rallying support, assessing local needs, and advocating based on citizens’ voices (23, 51, 52). Civil society can be a key ally in challenging corporate as influences, many movements campaign for stricter tobacco regulations, anti-obesity policies, and environmental protections. Expanding these efforts to hold businesses accountable for health harms is a crucial step forward (32). Civil society is more likely to succeed when it builds strong coalitions, aligns around evidence-based positions, and strategically leverages political opportunities (38). Our results highlight areas for improvement in civil society’s role in CPP. Some participants report a lack of involvement, a limited number of associations or a lack of focus compared to other areas of the cancer continuum. Addressing this issue could benefit from support from other sectors, such as the public sector, by providing a well-defined and transparent set of priorities or actively trying to involve civil society associations working in different areas. Some civil society associations lack expertise, a challenge that could be mitigated through collaborations with high-level institutions, such as those operating at the European level (52). Strengthening these partnerships would not only enhance their effectiveness but also increase civil society’s credibility and respect. Additionally, publishing reports could boost its visibility. Creating networks of CPP associations can amplify their influence, while collaboration with civil society institutions from other fields could further expand their resources and advocacy capacity.

While the results are presented separately for each sector, there are overarching issues relevant to all sectors. First, there are overlapping ideas between CPP and prevention of non-communicable diseases. Since tobacco consumption and dietary habits, among other factors, are risk factors for the development of both cancer and other non-communicable diseases (15), synergies may be formed with other public health initiatives focused on health promotion, broadening the scope of cancer prevention and avoiding confusion among the general public (15, 23, 53). However, this must be complemented by additional approaches, such as addressing infections or environmental exposures (53, 54). Second, participants from different sectors refer to social determinants of health (e.g., the need to adapt messages to different social groups, difficulties reaching vulnerable populations). Sectors must consider the potential of CPP to reduce cancer inequalities between socioeconomic groups, while also considering the differential distribution of risk factors and types of cancer incidence among social groups (21).

### 4.1 Iron curtain of cancer cases

Quantitative data suggests that Eastern European countries may rely less on the public sector for CPP than Western European countries, placing greater emphasis on other sectors. Qualitative data shows limited involvement from the public sector in Eastern countries, while the other sectors expect them to take the lead, set strategies, and facilitate the collaboration between partners. Results for Eastern countries highlight the need for available epidemiological data to assess the current situation and better define the potential gains from cancer prevention. This could help governments understand the cost–benefit of CPP and motivate citizens to advocate for measures. Eastern European countries (particularly the smaller ones) can also benefit from international collaborations, whether by joining EU projects or learning from the example of other countries. Small countries often face constraints not only in market dynamics but also in areas such as research capacity, policy implementation, and healthcare infrastructure. By observing how other countries have overcome these challenges, Eastern European countries can develop innovative solutions tailored to their own contexts. Finally, media and civil society can benefit from specific training to help them develop their potential as CPP stakeholders.

### 4.2 Merits and limitations

In this study, we were able to use a diverse sample of participants, from different countries, sectors, and scopes of activities. All participants were asked about their own activities as well as the role of other sectors, allowing for different points of view and avoiding the potential bias of a participant overestimating the work done by their own sector.

While the penta-helix provides a useful framework, classifying stakeholders into sectors involves some subjectivity. We minimized this bias by providing a standard definition of the sectors to participants in the survey and interviews; yet, we acknowledge that some bias may persist, as contextual and individual factors can influence how each participant interprets the definitions. Moreover, some stakeholders may be difficult to classify due to overlapping areas (e.g., non-profit associations representing private companies) or sector changes during one’s professional career. This overlap was also evident in the interpretation of some qualitative results (e.g., promoting workers’ health is the responsibility of employers, regardless of their private status).

English language was used in all data collection methods, even though the participants in this study were predominantly non-native speakers. This may have led to selection bias, as those willing to participate likely had a better command of the English language than non-participants and may have been more inclined to collaborate in international projects or read international scientific literature. While information bias could arise from difficulties interpreting the questions, we believe this bias is minimized, as points of agreement were found regardless of the participants’ proficiency in English.

Some participants were not exclusively dedicated to CPP or had difficulties identifying what was meant by the term (especially participants from the media). While a previous definition of CPP could have provided some standardization, the heterogeneity of responses allowed for the identification of information about general health promotion or secondary prevention that also proved relevant for the study’s objective.

Unfortunately, this study did not allow for a country-specific characterization of stakeholders due to the small sample size and the non-representativeness of the sample. Replicating this approach at a country-level would provide comprehensive knowledge useful for the design and implementation of country-tailored policies.

## 5 Conclusion

In conclusion, this study provides evidence that CPP goes beyond the National Cancer Control Programs. To unlock the full potential of CPP in reducing the cancer burden, appropriate interaction and complementarity between stakeholders from different sectors is essential. Insights from Eastern European countries perspectives offer a clearer view of the systemic and collaborative challenges affecting CPP, underscoring the importance of political stability and intersectoral partnerships for effective cancer prevention policies. Additionally, this study highlights the importance of multi-sectoral engagement in CPP, demonstrating that cancer prevention spans and influences various sectors. This is particularly true for commercial determinants of health, which play a significant role across sectors. Effective government leadership, responsible private sector engagement, independent scientific research, media transparency, and civil society advocacy are all crucial to mitigating commercial influences.

## Data Availability

The raw data supporting the conclusions of this article are available from the authors upon reasonable request, subject to appropriate measures to protect participant confidentiality.

## Group members of the 4P-CAN WP2 study team

Mirjana Babamova, Association for help and support of patients with gynecological diseases FEMINA M, Skopje, North Macedonia; Adriana Boată, Center for Innovation in Medicine, Bucharest, Romania; Stefania Boccia, Section of Hygiene, University Department of Life Sciences and Public Health, Università Cattolica del Sacro Cuore, Rome, Italy; Department of Woman and Child Health and Public Health, Fondazione Policlinico Universitario A. Gemelli IRCCS, Rome, Italy; Ana Cristina Garcia, Department of Epidemiology, National Institute of Health Doctor Ricardo Jorge, Lisbon, Portugal; Stanimir Hasardzhiev, National Patient Organization, Sofia, Bulgaria; Andreia Leite, Department of Epidemiology, National Institute of Health Doctor Ricardo Jorge, Lisbon, Portugal; Lilia Kriachkova, Dnipro State Medical University, Dnipro, Ukraine; Milica Kuzmanoska, Association for help and support of patients with gynecological diseases FEMINA M, Skopje, North Macedonia; Nikola Milasevic, Montenegrian Society of Medical Oncology, Podgorica, Montenegro; Vlad Nerau, The Bucharest University of Economic Studies, Bucharest, Romania; Ivaylo Petrov, Bulgarian Joint Cancer Network, Sofia, Bulgaria; Ramona Popescu, Center for Innovation in Medicine, Bucharest, Romania; Oscar Ribeiro, CINTESIS@RISE, Department of Education and Psychology, University of Aveiro, Aveiro, Portugal; Viktor Semenov, Dnipro State Medical University, Dnipro, Ukraine; Leonardo Villani, Section of Hygiene, University Department of Life Sciences and Public Health, Università Cattolica del Sacro Cuore, Rome, Italy; Department of Woman and Child Health and Public Health, Fondazione Policlinico Universitario A. Gemelli IRCCS, Rome, Italy; Ivana Vojvodic. Juventas | Leadership & Statistics & Management & Human Rights & Health and Social Protection; Clara Volintiru, The Bucharest University of Economic Studies, Bucharest, Romania; Rodica Milena Zaharia, The Bucharest University of Economic Studies, Bucharest, Romania; Serhii Zakharov, Dnipro State Medical University, Dnipro, Ukraine.
