# Supplementary material for: Characterizing stakeholders in cancer primary prevention in European countries: an exploration of challenges and opportunities using a penta-helix framework
Source: Front Public Health. 2025 Jul 16;13:1550712. doi: 10.3389/fpubh.2025.1550712 (PMC12307402; doi:10.3389/fpubh.2025.1550712)
Supplement: Supplementary file 1 [file Table_1.docx]

**Supplementary Files**

Table S1: Description of the penta-helix factors

| ***PUBLIC GOVERNANCE/ PUBLIC SECTOR​*** |
| --- |
| *All state institutions and organizations responsible for public administration and the implementation and management of government policies and programs.*  *Government and ministries, public health institutes and technical and scientific directorates (e.g., health directorate), etc.* |
| ***ACADEMIA/ RESEARCH*** |
| *Institutions involved in knowledge transfer to population and society; institutions that promote or carry out research in cancer field and strive to stimulate discoveries that benefit people, and to promote new standards of knowledge.*  *Universities, research institutes, foundations and scientific, expertise associations, etc.* |
| ***PRIVATE SECTOR​*** |
| *Sector of a national economy under private ownership in which the allocation of productive resources is controlled by market forces, rather than public authorities and other sectors of the economy not under the public sector or government.*  *Industries and any other profit-oriented organization; pharmaceutical industries, etc.* |
| ***MEDIA*** |
| *Media-based private or public institutions and organizations, and individuals that contribute to the “capital of information” within a particular social context.*  *Television, internet, newspapers, journalists, bloggers, influencers, and youtubers, etc.* |
| ***CITIZENS/ CIVIL SOCIETY*** |
| *All institutions or organizations that are public non-profit culture-based public. ​*  *Non-profit non-governmental organizations (e.g., Cancer Leagues), patients, or hospitals associations, volunteering, etc.* |

Table S2: Frequency of participants of the survey, by country and country group

| Country | n |
| --- | --- |
| Western Europe (n=48) |  |
| Austria | 1 |
| Belgium | 2 |
| Denmark | 4 |
| Germany | 1 |
| Greece | 3 |
| Italy | 14 |
| Malta | 1 |
| Portugal | 15 |
| Spain | 6 |
| Sweden | 1 |
| Eastern Europe (n=62) |  |
| Albania | 1 |
| Bosnia and Herzegovina | 1 |
| Bulgaria | 8 |
| Georgia | 1 |
| Kazakhstan | 1 |
| Lithuania | 1 |
| Montenegro | 12 |
| North Macedonia | 9 |
| Poland | 2 |
| Romania | 7 |
| Slovenia | 2 |
| Turkey | 1 |
| Ukraine | 16 |

Table S3: Interview guide

| ***A. INTRODUCTION*** |
| --- |
| *1. In which sector are you involved? [Share the slide with the sectors]*  *2. Could you please specify better how you are involved in cancer primary prevention - as a stakeholder - as an individual or on behalf of an institution?*  *3. What is the main institution where you carry out cancer primary prevention activities?* |
| ***B. ACTIVITIES*** |
| *1. In which main cancer primary prevention activities are you involved?*  *2. Are you satisfied with the cancer primary prevention activities you currently develop?*  *3. What are your main motivations for getting involved in cancer primary prevention activities?*  *4. Which barriers do you face when developing cancer primary prevention activities?*  *5. Which future activities would you like to develop?*  *6. Are you familiar with the European Code Against Cancer? And if so, have you been involved in initiatives promoting the European Code Against Cancer?* |
| ***C. PERCEPTIONS ABOUT OTHER STAKEHOLDERS*** |
| *We'd like to discuss the various stakeholders’ sectors involved in cancer primary prevention, specifically focusing on the public governance/ public sector, academia/ research, private sector, media, and citizens/ civil society.*  *1. For each sector, could you please tell us about the main differences in their activities and motivations? [public sector \| academia and research \| private sector \| media \| civil society]*  *2. Also, what do you see as the major barriers and opportunities for each sector in your country when it comes to cancer primary prevention?*  *3. Of all the mentioned sectors, in your opinion, which sector has the main role in change concerning cancer primary prevention in your country? Why?*  *4. In your opinion, which sector is the most proactive in addressing cancer primary prevention in your country? Why?*  *5. In your opinion, which sector is the most influential in addressing cancer primary prevention in your country? Why?* |
| ***D. INTERACTION BETWEEN STAKEHOLDERS*** |
| *1. With which main partners (institutional or individual) do you collaborate when you develop cancer primary prevention activities? What is the main focus of that collaboration? What could be improved? Have you had bad experiences collaborating with other partners?*  *2. How do you characterize the collaboration between sectors in your country? Which entities best interact together to address cancer primary prevention in your country? What could be improved in the collaboration between entities?*  *3. Finally, we would like to know if is there anything else you would like to add on this topic that you feel might be significant and not mentioned during the interview?* |

Table S4: Additional quotes from public sector

| ***A1. LOOKING AFTER CITIZEN’S HEALTH*** |
| --- |
| **Activities**  Complexity of players: *“Health Insurance Fund has to finance this screening and this screening program, not to be in the Ministry of Health, but to be part of the contracts that have Health Insurance Fund with the gynecologists in the primary level”*  Power: *“they have the tools and they have the power”*  Strategic role: “*They work together with other specialists to elaborate this national cancer plan”* |
| **Motivations**  Responsibility and impact: “*the public sector should have a strong motivation to be involved because of their essence, of their core business, that is to protect the public, the population. From harmful behaviours, harmful products”*  Tackle burden of disease: “*In the 21st century, everything has to push us to the cancer. Everything, food, air, everything. It's really dangerous”* |
| **Barriers**  Political circumstances: “*we have really frequent changes of government. So even when you are starting some kind of a project, you know, there comes a different government as you are starting from scratch because that government cannot see the value. And there we have the most, that's maybe the biggest barrier because we don't have continuity in the project”*  Threats to power: *“whenever we translate a scientific decision or scientific knowledge into legislation and if this knowledge has a conflict to the economic stakeholder, for example, the industry, tobacco, alcohol, or even sunburn, there is a barrier that must be considered”*  Prevention not a priority: *“So, most of the money goes to treatment, because it's very expensive. And treatment, when I say treatment, is surgery, radiotherapy, chemotherapy, and so on. And a very few percentage of the money goes to primary prevention”*  Institutional barriers: *“Sometimes we have the economy ministry that do not want people to stop smoking because they need the taxes or the agriculture ministry, they don't want to take measures against the wine consumption or beer consumption. So there is a conflict of interest between the different ministries”* |
| **Opportunities**  Pivotal role in change: *“It's obvious maybe that the health authorities can change regulation and they can change the things, the way that things work”*  Have a wider scope: *“just supporting more projects, more networking with other sectors, more external cooperation, bringing people with experience from abroad”*  Gains in the long term: *“if the government could allocate, or could increase the proportion of money that goes to primary prevention, I think in 10, 20 years, we could have a better, more healthier population”*  Cooperation/ involvement: *“the government, the public governance, of course. Because they are the ones that have, should have, the strength to change things and we know that, for instance, for the reduction of tobacco consumption, we have to have multiple agents working, not together, but at the same time or coordinated”*  Political opportunities: *“the ministry of health is aiming high. They have a lot of plans. good step forward and they are making changes”*  Become more aware: *“Because if there weren't any, any national campaigns, for instance, from the National Cancer League at different levels to widespread the message that tobacco is bad for you, when the government tries to pass restrictive laws, they wouldn't even try it”* |
| ***A2. MAKING THE SYSTEM WORK*** |
| **Activities**  Epidemiology: *“And I gathered the data from the sources, from the hospitals, from the laboratories, regarding the hospitals, both the public and the private hospitals”*  Study what works: *“We made piloting with HPV testing on the clinic of our university clinic of gynecologists”*  Provide structure: *“How to manage, how to identify the potential patients, how to identify potential providers/ health providers, how to easily access the women to the health services, especially gynecology services”* |
| **Motivations**  Improve the system: *“So, if we can prevent this from happening and there are fantastic tools for it in the past 20 years, if we can implement a nationwide implementation of measures to prevent future patients, it's a great help. And we can participate in this, it's to translate scientific knowledge to public application, to public legislation and regulation, so it's an opportunity to bring some science from the ground to the back stage and to the policy makers”* |
| **Barriers**  Structural issues: *“We don't have universal health coverage here.”*  Insufficient data: *“the data that they used to describe the physical activity level that [country] has, the number of healthy and not-so-healthy foods that we have, the tobacco consumption, the alcohol consumption are all reports from at least five years ago.”* |
| **Opportunities**  Create networks of professionals: *“to form a community and to train some personnel, maybe like medical nurses”*  Write procedures: *“We have to make all these rules on paper”*  International cooperation: *“this is a good example from Slovenia. We have a similar health system, and also we are the same population.”* |
| ***A3. OPERATIONAL ENGAGEMENT*** |
| **Activities**  Health education: *“I work on cancer screening and so we often use the setting of the cancer screening into our hospital or outpatients to promote good behaviours and all the behaviours that can be useful to us against cancer development”*  Do screening programs: *“the Ministry of Health, with all the side organizations, they are calling women by the phone”* |
| **Motivations**  Educate the population: *“So I hope that the motivation is the same for all of them. How can we decrease the prevalence of cancer and how can we avoid cases. And how can we educate the population on how to manage the risk factors”* |
| **Barriers**  Lack of money: *“the funds for primary cancer prevention are simply not there, they are not budgeted, money is not available and therefore the activities are not implemented”*  People-related barriers: *“the lack of literacy in our public target because people don't have enough literacy, health literacy to sometimes understand the need to have some kind of lifestyles or promote what they can do to prevent diseases and to really understand what that means.”*  Difficulties implementing: *“This plan is ready from the January 2023, but there are no activities about this plan. They are not ready”*  Inequalities: *“The government has money, and they have the power to create legislation and regulations and so on, but they are not close to the people”*  Human resources: *“I was constantly asking my supervisor to hire more people for the registry, and that never happened”* |
| **Opportunities**  Deal with hierarchy: *“my experience is whenever there's a good will from the mid-level and low-level management, things are doable and fully implementable.”*  Extend the scope of activities: *“Maybe we should implement some moving programs, like maybe I have an idea that maybe the city or the government will give some money to some sport clubs, fitness clubs, like to pay for the patient who can't afford it to come into the fitness area”* |

Table S5: Additional quotes from academia/research

| ***B1. SCIENTIFIC CREDIBILITY*** |
| --- |
| **Activities**  Trustworthiness: “*because when information is coming from academia, they normally encounter a higher level of acceptance”*  Knowledge creation: *“Provide knowledge. conducting research to understand better diseases in general. […] of cancer, but also prevention and so.”*  Scientific support to others’ activities: *“everything is based on scientific consensus regarding primary prevention of cancer.”* |
| ***B2. DIVERSITY OF APPROACHES*** |
| **Activities**  Basic science: *“to conduct basic research in the lab, that impacts also all the kind of downward analysis and research”*  Characterize the situation: *“with this study, we estimated the exact amount, the exact number of cases and deaths attributable to tobacco smoking.”*  Innovation: *“We have developed some specific tools, like smartphone apps”* |
| **Motivations**  Raise awareness to a difficult situation: *“So we just thought it would be a good basis to see where we are. And the results are so devastating. And so they can serve as a good fundament, as a good basis to see where we stand.”*  Lack of data: *“Because at that time, there were no data about incidence or survival.”* |
| **Barriers**  Financial barriers: *“I would like to do some researches that would involve, of course, some prospective studies and do something related to, you know, to this. But financial barriers are number one.”*  Human resources: *“it's very hard to gather a team, because everybody is so engaged in the clinics, you know, work with patients. So, the research segment is something that is an additional burden to those people working at clinics, dealing with patients and stuff like that. It's very, very hard to gather a good team. So, it's very easy to sign something, yes, I'm in, but after the project starts, we're usually faced with so many difficulties and everything just ends up on the shoulders of two to three people and it's not enough to go, you know, further with projects and to carry out some good results.”*  No clear direction: *“we are navigating without the compass in this area”*  No resources/ no equipment: *“Equipment, of course, so we're lacking in some serious equipment when it comes to basic research, especially in cancer.”*  Limited scope: *“it's not a very explored area in that sense, so we do not have many groups working on research on this type of areas. I'm not saying that we are the only group because we aren't, but there's not many people out there doing research on this type of things in a systematic way.”* |
| **Opportunities**  Expand the scope of research: *“we should actually develop different tools and different ways of trying to tackle this and trying to convey this type of information and change things.”*  Multidisciplinary/ integration: *“I think the main mission for them is to make it very interdisciplinary”*  International cooperation: *“because we have the access to EU projects, EU partners, that's why we are maybe more advanced than the rest of the country”*  Increase support resources: *“if enough resources were put into primary prevention, the research and academic world would implement more research on primary prevention.”* |
| ***B3. GETTING OUT OF THE LAB*** |
| **Activities**  Teach/ train: *“Educate doctors. to educate the new generation of doctors”*  Write guidelines: *“I helped with writing the new standards for primary prevention”*  Education at schools: *“sometimes, not often, I'm invited by, for example, charities around, and I'm going into school, the companies and so on. To present what should we do to prevent the emergence of cancer”* |
| **Motivations**  Empower people: *“We are always trying to convey not only information, but ways or tools so that people can empower themselves and change behaviour towards a more healthy behaviour in the sense that by doing this we are reducing the risk of cancer at a population wide scope. So, the idea is to find different ways of promoting these types of behaviours”*  Get down the cancer/ current state: *“It's the state and the condition that my country is at this point. we need to start somewhere, right?”* |
| **Barriers**  Isolation/ no collaboration: *“Academia is for academia, you know, it's for academia, it's not for normal people. For citizens and normal people, it's not approachable, because we don't know what they are talking about. So, academia is for academia, you know, and it's not practical, it's theory, and we have problem that theory to provide in practice. I'm not a doctor, you know, and they are in theory, and the theory has to be the basics from the practice”* |
| **Opportunities**  Make research more visible: *“They do it already through the foundation, but not always through directly academia, university to the media. Sometimes, but not always, not enough. They could publish more studies about the research they do in order to get down the cancer”* |
| ***B4. LIFE IN ACADEMIA/ RESEARCH*** |
| **Activities**  Academic cooperation: *“This one is regarding personalised primary prevention and the second one is with [international partner] and this one is regarding screening personalised screening because these countries, they are at this point the most developed regarding screening programmes”*  Step in the career of people from other sectors: *“very involved in academic research activities for the dissemination of our research or of our results.”*  Meetings/ networks: *“we created the network of expertise. create training opportunities between different cancer centres”* |
| **Motivations**  Scientific interest: *“the great motivation is really to know more about cancer, to know more about how it can be prevented primarily”*  Get funding: *“the way investigation was funded, is funded, shapes the landscape of research, cancer research in the country”*  Contamination between areas: *“I completely moved, shifted to the human side”* |
| **Barriers**  Methodological aspects: *“you need the large population for a long time to do a really properly done prevention study in the long term... is fairly complicated and very expensive”*  Journals not interested: *“Because we really we really need the impact and I know education, especially at population level, it takes a lot of time”*  Difficulties finding financing: *“funding is not directly for this type of projects. So, you have funding for basic research. Yeah, and that's one of the main areas of funding that we have, we have some funding for clinical trials and other stuff. But this middle ground, this area that’s a little bit grey in terms of research because we are trying to do intervention research. And in an area that's health related but not medical, per si”* |
| **Opportunities**  Leave the bubble: *“It's only by the academia bubble that they can make it, I don't know more regulation or fix it or working together in order to create a system that is much better, good for the patient in the end of the day.”* |

Table S6: Additional quotes from the private sector

| ***C1. PROFIT-ORIENTED*** |
| --- |
| **Activities**  Development of products/ innovation: *“The private sector is good because they provide these tests”*  Activities dependent on their objectives: *“with the population in a whole, or sometimes in a specific population”* |
| **Motivations**  Profit: *“They are commercial companies and they need in the end of the day to have a positive result in financing”*   - Limited interest: *“But I still believe that it is a bit of a contradiction when they do so, because in principle, that is not their core business”*   Marketing and image: *“don't know if any industry is actually working on cancer prevention as a theme, I think that in terms of pharmaceutical industries, they are more concerned on conveying an image that they care and not actually doing anything particular about this”*  Own agenda: *“they will sponsor and they will finance some doctor to go to the, I don't know, to some symposium, some training. And then when the doctor came in our country, they will promote only this product”* |
| **Barriers**   - Focus on profit: *“it's very hard to start collaboration with private sector because prevention is a sector that has no economic impact for private industries or private organizations in a short time.”* - Insufficient involvement: *“I don't see really a role of the private sector in promoting that prevention. If it's really not aligned with their own goals.”*   Lack of return: *“Sometimes the private sector don't have that kind of return they expect. So that may be a barrier regarding further research on that specific field or on a specific vaccine.”*  Limited scope/ inequalities: *“if they don't have the knowledge, then it's difficult that they develop the willingness”* |
| **Opportunities**  Clear message/ build their reputation: *“we need to become visible and show that we have a good reputation so that if they link their activities on social responsibility”*  Building the relationship/ Red lines: *“we have to have some red lines and to have a very good framework how to work with them”*  Regulations: *“if they want to fund us, the activities that we consider to be beneficial for our patients, yeah, that collaboration will happen, but we will not collaborate if we are imposed to do something because at this point it becomes illegal and it is illegal. More regulations regarding private sector.”* |
| ***C2. RESOURCES AND OPERATIONAL ACTIVITIES*** |
| **Activities**  Providing resources: *“there is a lot of projects that are happening with the support of the private sector and they are involved in a lot of this”*   - Support others’ activities: *“pharmaceutical industry is mostly, mostly involved in supporting of non-governmental organizations during their activities”*   Raise awareness: *“So basically, practically, what we are doing is education and raising awareness. If I can put all these activities in that scope.”*   - Vaccination/ screening: *“we have the campaigns about vaccinations here. Also, the HPV vaccination was part of this. So yeah, we support HPV-based screening”*   Capacity to reach people: *“always the private sector has the best efficacy to communicate and to get reactions on social media, on television. And they can cover the most part of the population because they are very effective.”* |
| **Motivations**  Educate and promote health of people/ change mentalities: *“I don't know how much you know about [country], but here the mentality is a little bit, I don't know if you have a symptom, like you are hiding the symptom.”* |
| **Barriers**  Bad reputation: *“I think in [country] we have a problem because we look at the private sector as an evil thing”*   - Conflict of interests: *“For instance, private sector, specific pharmaceutical industry, sometimes it's not so easy to have collaboration with the public governance due to conflict of interest”*   People’s beliefs: *“after COVID and after all that's happened around COVID and all the vaccines and everything, there is a rise in skepticism about the vaccination”* |
| **Opportunities**  Expand activities/ think outside the box: *“Start with children. So it's how we educate the population. And we should also start with not only adult population, but with children.”*  Geographic and sociodemographic outreach of pharmacies: *“you're reaching better the whole population […], pharmacies reach all population”*  Pharmacies’ collaboration with research: *“we can collect a very big amount of data”* |
| ***C3. ETHICS AND RESPONSIBILITY*** |
| **Activities**  Promote the health of the employees (included in general health promotion): *“Yes, so the activity that we are doing as a company, they really span from, let's say, primary prevention. So, for example, looking at all the habits for food and drinking and physical exercise. So, there are regular information campaigns reaching out to all the employees about this. “* |
| **Motivations**  Personal reasons: *“Due to my job, I heard so much amazing stories of fighters, of everything. I was so much inspired by them to do a change. So, that's my main motivation in life generally, not in my job, but in life generally. I, you know, when you do a good deed, you feel proud. You feel good. Nobody has to know, but you feel good.”*  Capacity to prevent cancer: *“although not all cancers are preventable, at least 40 percent of the cancers are preventable”*  Ethical concern/ responsibility: *“there could be some with an interest and the interest could be an ethical ethic interest. And to build up their responsibility, social responsibility, reputation, and ethical reputation.”* |
| **Barriers**  Pressure on governments, pressure on citizens: *“They put pressure on the government by pressuring citizens. The main barrier to me there is essentially the industry. So, like, for instance, the tobacco industry or the alcohol industry, the food industry, putting pressure on the public sector with all sorts of arguments, such as that they help the economy or they start with saying that their products are not harmful, that people should be free to choose, but they influence the public sector in a negative way.”* |
| **Opportunities**  Be responsible selling products: *“Large impact if they did not exist. If the industry did not exist, or like if the food industry, Coca-Cola didn't sell Coca-Cola, then we didn't have to advocate for the harmful, or if the tobacco industry was made illegal, then that would help a lot… tobacco, I mean, primary prevention”*  Companies as a concentration of people: *“the company are self-started, they do by themselves, but the employee, they're also citizen ultimately. So I think it will be nice if, for example, from the government standpoint, they think about the screening campaign also in this way. So, leveraging employers, because in company you have concentration of people.”* |

Table S7: Additional quotes from media

| ***D1. CAPACITY TO REACH PEOPLE*** |
| --- |
| **Activities**  Great impact: *“There is no month without a cancer debate on different subjects, treatment, education, screening, prevention. And I think because of this discussion, media can send a lot of messages and they are very influential of what we do, because we cannot reach so many levels like the media”*  Place to influence people/ policy: *“When the problem became public, that means that the ministry will resolve it, and when the problem is secret and no one knows, the problem will remain the problem. it's always the best thing is to say, yes, we have the problem, when we have the problem, when the media publish the problem, the government will try to resolve the problem.”*  Create awareness: *“And then we can, we can build the documents, the strategies, the programs, the plans, everything. But to take the plans and the programs and the strategies to the common citizen and make the common citizen become aware of what he or she can do to promote their health and to prevent diseases.”* |
| **Motivations**  Pressure policies: *“Education in this field is very, very important. In the end, not necessarily the last, from the important point of view, is that maybe we can put some pressure sometimes when politicians and politics are not following the needs, the societal needs”.*  Public service: *“the media should do a public service, so a service to the population. And in that respect, working on primary prevention, promoting primary prevention would be essential from the media perspective.”*  Dubious motivations: *“And not every time it's happening this. And sometimes these things make media motivated to send the wrong message, because they are gaining more money in publicity, because there are more people that see the message. And it's dangerous”*  Commercial interests: *“And it's a wrong message, of course. Some influencers say they don't work and you shouldn't use them. It's for publicity, but it's a wrong message”* |
| **Barriers**  CPP not a priority: *“it's just that also a lot of doctors involved in cancer primary prevention do not have enough time to show on media, and even if they show those, you know, time slopes for their showing, it's just early in the morning when nobody listens to this kind of story and stuff like that”*  Influence by politics: *“our media sector is very under political influence”*  Compliance with news cycles: *“media are covering the trends and media are covering special awareness months, special projects”*  Bad reputation: *“they think that we are sharks, we are not, we are just trying to help patients and to make the problem public, you know”*  Conflicts of interest: *“I had bad experience with media. Making some news about something that I said that was not true. So even without the patient authorization, I had bad experience sometimes”* |
| **Opportunities**  Powerful tool: *“I think in our country, and based on the mentality and the health awareness of our citizens, media could be the powerful tool for, you know, enhancing cancer primary prevention, but they're not doing it”*  Adjust the dialogue: *“Learn how to talk to them. Because you need to know how to talk to them, and this is really not something that you learn easily”*  Choose the best channels: *“do we use radio as a channel for this target population? It cannot be one-size-fits-all, that's my point.”*  Impact policies: *“to see if the public policies would change to secure people with cancer”*  Two sides of the story: “*Because we don't have to, we can't, and it's not normal to write everything that patients said to us, because patients are, when patient is unhappy, so he will tell us anything, but I can't write everything, so I always ask some ministries, institutes or something”*  Public perception change: *And the idea was to partner with [journal] and to create a positive impression of people that have already cancer* |
| ***D2. DIVERSITY AND SCOPE*** |
| **Activities**  Different channels, including new media: *“So also depending on the target population, depending on the age, some people listen more to radio, some other to television, some other don't trust the radio or television, but they go to the stadium. So, it should really be stratified.”*  Diversity of topics and activities: *“We cover extensively cancer topics, different kind of topics, starting with the science of cancer, but also in terms of politics, in terms of, I don't know, regulations, programs, European national programs, prevention, treatment, and so on. So, health and medicine”.* |
| **Barriers**  Channels not trustable: *“the capacity for misinformation is very high, so there's lots of misinformation associated with cancer prevention. we see it in many places, Youtube, and other type of social media is more keen on doing this. But even in newspapers we see it.”*  Sources not trustable: *“It's just that they have an experience, they have an opinion. Everyone, every citizen has their own opinion, but not every citizen are writing their opinion. And it's very, very risky to send this opinion in a public, because we saw now that we have a problem with our patients, or we have a problem with our population in anti-vaccine, anti-health system, or doctors. Because they see on Dr. Google, and on Dr. Google it's everyone, it's influencers, it's doctors that are anti-vaccine, it's everything over there. So, this is a very risky thing.”*  Online commentaries: *“The only sad experience I can have is about the commentary, because we publish on all platforms, and the people, our readers can write commentary. And the commentary is sometimes are bad, because of the pharmaceutical society, they say that to make money, it's like a bit complicated”*  Lack of in-depth focus: *“There are some other colleagues who are covering in the national channel, but they do shorter, much shorter things”.*  Too many scopes: *“So I think there is a balance between the knowledge sharing that we do at an EU level, the knowledge sharing that we are doing for [countries], but then also the knowledge sharing that we are doing at a global level on cancer prevention research, just because the science, the data, the national policies on risk factors, and the prevalence of cancer differs based on GDP, based on healthcare services, based on healthcare budgets.”* |
| **Opportunities**  Visibility given by new media: *“because I'm seeing here the bloggers and the influencers and the YouTubers, so we know about the harms of social media nowadays, but if these bloggers and influencers and YouTubers, if they can use their stories, for example, people who have cancer and who decide to be a blogger or an influencer or a YouTuber, to tell their story and to influence positively others to take care of their health, I think this is something that these people can use their stories for the common good”* |
| ***D3. INFORMATION AND DISSEMINATION*** |
| **Activities**  Provide information: *“You can get access to information very quickly”*  Disseminate specific news: *“They are giving us information when they are doing some kind of public activities, campaigns and everything”*  Interaction with other sectors: *“We have to always ask stakeholders like ministry or hospitals or NGOs that are taking the fight with cancer or prevention of cancer in their work area”* |
| **Motivations**  Educate the public: *“it is important to know the science of cancer, to say so, to know what you can do to prevent”.*  Responsibility: *“Also, being the public radio, we have this mission, and I would say obligation to inform the listeners, to inform the general public. Public radio has a national coverage”.*  Personal experiences: *“I’m a woman, so I have many women friends. And through all these things, I know that women don't go to doctors to get the preventive check-in exams.”*  Interesting topic: *“It's interesting, of course, from the scientific point, from the research point of view, it's absolutely amazing the progress that was made during the last decades”* |
| **Barriers**  Responsibility not fulfilled: *“sometimes they lack responsibility”*  Unprepared journalists: *“very small amount of journalists that are, can do the texts and articles about cancer and health, you know, because you have to know”*  Low quality of information: *“Just yesterday, I saw an article, a press conference article about Sunscreen because some bloggers, some influencers in [country], and I think it's because they send wrong messages saying that sunscreens are not... It's not necessary. They don't serve, they don't work.”*  Lack of resources: *“Well, speaking for myself, I would like to be able to do more in terms of having more, I would say, physical resources. I'm only one”.* |
| **Opportunities**  Adjust the messages: *“you need to find what is exciting for the public”*  Send the right messages: *“somehow checking on the private advertising if it is really not too much outrageously pushing some compounds”*  Training for journalists: *“So journalists also have to be educated how to report to public regarding disease awareness”* |

Table S8: Additional quotes from the civil society

| ***E1. PROXIMITY TO PEOPLE*** |
| --- |
| **Activities**  Participation of persons from other sectors: *“I do some volunteering with the Association Against Cancer, typically in breast cancer.”*  Personal involvement: *“So we are involved also as an organisation, as a civil society, but also as a person interesting in this subject, a citizen”.*  Proximity to people: *“grassroot activities, starting the discussions to get patient perspectives heard at meetings, for example, at the UN level or the European Commission level. But I guess it's the starting point, the foundation of grassroots mobilization of research data and lived experiences of patients, I guess.”* |
| **Motivations**  Personal involvement besides one’s job: *“I will set an example from my kids, from my family and from the people close to me.”*  Personal experience: *“My 1st motivation is that I have in the family a patient with cancer, so I know about this. This is the first experience having in the family.”* |
| **Barriers**  Lack organization/ lack strength/ lack power: *Cancer patient associations, they are very scattered and they are fragmented. So they have their several agendas. And sometimes it would be more profitable and resource optimized action to concentrate their effort towards the same goal.*  Disregard by others: *“There was this physician that I knew, who once called the [institution] where I worked, and he was complaining that I was writing about cancer, and I was not the physician.”*  No expertise: *“Because the private, the NGO sector is a sector without experts”*  Stigma/ cultural issues: *“when speaking about altruism, [country] is on one of the lowest ranking countries in Europe and in the world when it gets to the altruism, […], we're on the true bottom of the list.”*  People-related barriers: *“you also see these fatalistic reactions like, I don't want to know, I don't want to”* |
| **Opportunities**  Changes in structure: “*it would be nice to have a patient representative who is actually a lawyer”*  Joining forces: *“they don't have the strength they could have if they joined efforts and built a national federation for oncological patients”*  Advocacy/ strength to change things: *“Cancer League can help academia research because they can record money and they can convince people and have money for more research and more sensibilization. So, they can help with public environment and academia and research all together”* |
| ***E2. ADVOCACY AND VOICE*** |
| **Activities**  Influence and advocacy: *“other nonprofits that are trying to move forward and push forward some policies that can help people prevent diseases. pushing the agenda”*  Personal history as their voice: *“but to bring their stories and their opinions and also their experience to contribute when everything that they know, the citizens, especially those that went through a disease like cancer”*  Central part in the health system: *“They are always heard, and nowadays there are these patient advocates and the patients themselves, they have a very strong voice in where the money goes, and I think this should be one of the main motivations of citizens, is to force the government to use wisely our money that we pay, our taxes, and to apply this money wisely and based also on scientific consensus and not on ideology”* |
| **Motivations**  Burden of cancer: *“So I think we need to have stakeholders that work in the area to make it more, i don't know, well known about the prevention and about the cancer because the cancer affects a lot of the population, especially here in [country]”*  Raise awareness: *“The main motivation is to sensibilize the most people as they can”*  Inefficiency of the government: *“it's better to do something like personal activity compared to the waiting for the government to organize something.”* |
| **Barriers**  Lack of awareness: *“Even when, even if and when organizations or civil society organizations realize the importance of prevention, primary prevention, then they cannot really be active in primary prevention, because there is a lack of resources, human and financial”*  Insufficient involvement/ not a lot of patient organizations: *”Not a lot of patient associations (stigma and survivors do not want to talk about it). if you look at the patient association that might be here, also because, yeah, organizations that are non-profit, so patient associations can be here. You don't have many related with cancer. They just want to live”*  Isolation/ no visibility: *“the last letter is just the bottom of all these stakeholders, because civil society, they're not very well, I mean, there are some, you know, non-governmental organizations who are into it, but they're just not present enough in media, and so, basically, if you have cancer, I'm not talking about primary prevention, it is usually your own problem, and the problem of people who are experiencing the same problem”* |
| **Opportunities**  Expand activity: *“we can do is to train journalists on the importance of primary prevention. I think that is very important to educate them”*  International cooperation: *“So we have tried so many times to connect these people with patient organizations from other countries, but with no success”*  Fill the needs: *“And I think we need more and more civil society to be involved in order to decrease the needs of our population, the needs of information, the needs of education, the needs of accessibility to different programmes or different doctors”*  Opportunities to educate/ raise awareness: *“in the future, I would like to continue this work because, especially for adolescents who are just picking up tobacco smoking, I think they are still very open to people outside of their reality. Probably they don't want to listen to their parents, but they will listen to people who are working actively in the field and who know how to talk about these things”* |
| ***E3. DO WHAT OTHERS DO NOT DO*** |
| **Activities**  Education/ awareness: *“I saw that it's more important to educate people, to organize them to be active in this important self-care. Start from the day one to educate people, to explain, to do different information campaigns”*  Filling the gaps: *“We work more than eight years about the national cancer plan. It's correct to write an international cancer plan. Now our activity is to activate this plan”*  Extensive scope of activities: *“I know that they work tirelessly, they work tirelessly and they don't work only on breast cancer screening on the mainland. They work across the board. They work in screening, they work with primary prevention”* |
| **Barriers**  Lack of resources: “*the activity of the civil society is also strongly depending on the availability of the funds”*  Limited scope: “*I have the impression that people that are more active in civil society are also the people that are easier to be involved in a good way if we perform lifestyle changing programs. So I think that the good intention of civil society has not the capacity to reach the people that could have”*  Overlapping services: “*there are NGOs overlapping the same services in the municipality”* |

Table S10: Number of interviews by sector, country and country group

|  | Western Europe | Eastern Europe |
| --- | --- | --- |
| **Sector** |  |  |
| Public Governance/ Public Sector | 2 | 5 |
| Academia/ Research | 2 | 3 |
| Private Sector | 3 | 5 |
| Media | 2 | 3 |
| Citizens/ Civil Society | 3 | 5 |
| **Country** |  |  |
| Belgium | 1 | - |
| Bulgaria | - | 6 |
| Ireland | 1 | - |
| Italy | 5 | - |
| Montenegro | - | 5 |
| North Macedonia | - | 3 |
| Portugal | 5 | - |
| Romania | - | 4 |
| Ukraine | - | 3 |

Table S11: Characterization of the interview participants, by sector and country group

| **Public sector** | *Western countries*  - Officers in a public health institution (national)  - Team members in local health units  *Eastern countries*  - Officer in a public health institution (national)  - Officer in public health institutions (national and cross-national)  - National representative in an international health-related organization  - Health-related technician  - Family doctor in a local health unit |
| --- | --- |
| **Academia/ research** | *Western countries*  - Researcher (CPP implementation)  - Professor and researcher (cancer)  *Eastern countries*  - Researcher (cancer epidemiology)  - Project-manager and consultant (CPP projects)  - Professor and researcher (cancer clinical research), interested in CPP |
| **Private sector** | *Western countries*  - Representative in a pharmaceutical company  - Representative in a pharmaceutical company  - Officer in an association representing community pharmacies  *Eastern countries*  - Representative in a pharmaceutical company  - Representative in a pharmaceutical company  - Representative in a pharmaceutical company  - Officer in an association representing pharmaceutical companies  - Officer in an association promoting innovation in health-related companies |
| **Media** | *Western countries*  - Journalist (web), interested in CPP  - Officer in a communication company dealing with cancer-related projects  *Eastern countries*  - Journalist (web, radio), interested in cancer and health issues  - Journalist (journal), disseminating news to health professionals  - Editor (newspaper), interested in cancer |
| **Citizens/ civil research** | *Western countries*  - Representative of a cancer-patient organization  - National representative of an European-level patient association  - Former officer in a National Cancer League  *Eastern countries*  - Representative of a cancer-patient organization  - Representative of a cancer-patient organization  - Representative of a cancer-patient organization  - Representative of a cancer patient organization  - Private citizen with special interest on CPP |
